# A critical comparative study of the performance of three AI-assisted programs for bone age determination

**DOI:** 10.1007/s00330-024-11169-6

**Published:** 2024-11-05

**Authors:** Johanna Pape, Maciej Rosolowski, Roland Pfäffle, Anne B. Beeskow, Daniel Gräfe

**Affiliations:** 1 Department of Pediatric Radiology, University Hospital, 04103 Leipzig, Germany; 2 Institute for Medical Informatics, Statistics and Epidemiology, Leipzig University, 04107 Leipzig, Germany; 3 Department of Pediatrics, University Hospital, 04103 Leipzig, Germany; 4 Department of Diagnostic and Interventional Radiology, University Hospital, 04103 Leipzig, Germany

**Keywords:** Bone age, Artificial intelligende, Multivendor, Comparative study

## Abstract

**Objectives:**

To date, AI-supported programs for bone age (BA) determination for medical use in Europe have almost only been validated separately, according to Greulich and Pyle (G&P). Therefore, the current study aimed to compare the performance of three programs, namely BoneXpert, PANDA, and BoneView, on a single Central European population.

**Materials and methods:**

For this retrospective study, hand radiographs of 306 children aged 1–18 years, stratified by gender and age, were included. A subgroup consisting of the age group accounting for 90% of examinations in clinical practice was formed. The G&P BA was estimated by three human experts—as ground truth—and three AI-supported programs. The mean absolute deviation, the root mean squared error (RMSE), and dropouts by the AI were calculated.

**Results:**

The correlation between all programs and the ground truth was prominent (*R*^2^ ≥ 0.98). In the total group, BoneXpert had a lower RMSE than BoneView and PANDA (0.62 vs. 0.65 and 0.75 years) with a dropout rate of 2.3%, 20.3% and 0%, respectively. In the subgroup, there was less difference in RMSE (0.66 vs. 0.68 and 0.65 years, max. 4% dropouts). The standard deviation between the AI readers was lower than that between the human readers (0.54 vs. 0.62 years, *p* < 0.01).

**Conclusion:**

All three AI programs predict BA after G&P in the main age range with similar high reliability. Differences arise at the boundaries of childhood.

**Key Points:**

***Question*** *There is a lack of comparative, independent validation for artificial intelligence-based bone age estimation in children.*

***Findings*** *Three commercially available programs estimate bone age after Greulich and Pyle with similarly high reliability in a central European cohort.*

***Clinical relevance*** *The comparative study will help the reader choose a software for bone age estimation approved for the European market depending on the targeted age* group and economic considerations.

## Introduction

The Greulich and Pyle (G&P) method remains the most common method for determining bone age (BA), and it involves the comparison of the X-ray image of the left hand with reference images [1, 2]. Compared with other BA determination methods, G&P can be easily implemented into clinical practice, but it depends on the reader’s experience, takes time, and shows considerable inter- and intraobserver variability [1, 3, 4].

Several programs based on deep learning have been able to circumvent these limitations in recent years and provide an estimate of BA within seconds comparable to that of expert readers without relevant intraobserver variability [5–9]. AI (Artificial Intelligence)-assisted BA assessment aims to reduce the dependence on human input and provides a better alternative to the subjective traditional BA assessment [10, 11].

Four commercially available programs for determining BA using the G&P method are currently admitted for medical use in Europe by Conformité Européenne (CE): BoneXpert (Visiana)—since March 2009; VUNO Med-BoneAge (Vuno)—since May 2018; IB Lab PANDA (ImageBiopsy Lab, Vienna, Austria)—since November 2020; and BoneView Bone Age (Gleamer)—since March 2023 [12]. As the first commercial, CE-certified AI software for automated BA determination, BoneXpert, was launched in 2009 [9] and determines the BA of every single bone of the hand and wrist by analyzing shape, intensity, and texture. The overall BA is calculated by taking the aggregate of the individual values [13]. BoneXpert was developed as an “autonomous reader,” completely replacing a human reader. In contrast, the CE-certified AI software PANDA and BoneView were developed to assist the human reader in clinical practice. In practice, images and results still must be checked by a human reader using the G&P atlas.

Besides internal validation as part of CE certification, independent external validation is particularly important for assessing the real-life performance of AI-based programs. To our knowledge, all AI-supported programs for BA determination have been validated separately, except for one study comparing two programs on the same data [14].

Each of the performed validation studies provides quality parameters, such as the mean absolute deviation or the root mean squared error for BA estimation, which potential buyers and users can use to estimate the performance of the software. However, the differences between those validation studies regarding age, ethnicity, and population size, as well as the varying expertise of the G&P expert readers representing the reference standard, render the comparison between the different programs less objective.

Thus, the current study aimed to compare the performance of three AI-supported programs for determining BA in a single collective of Central European Caucasians.

## Materials and methods

### Patient cohort and selection

A patient collective already described in another context was used for this retrospective study [15]. It comprised patients aged 1–18 years who had received an X-ray of the left hand for clinical BA determination between 2011 and 2020. All images were taken at a Central European tertiary center within an institute of pediatric radiology. The radiology information system was used to identify 5612 images. After categorizing all patients into 1-year age groups, nine male and nine female patients were randomly selected for each group, resulting in the inclusion of 153 male and 153 female patients, as published in Gräfe et al [15] (Supplementary Fig. 1). A positive ethics vote of the local ethics committee was obtained for the retrospective analysis of the images and informed consent from the patients was waived because of the retrospective study design (EK 46/2020).

### Subgroup for the most common age range

In infants and older adolescents, BA is rarely determined in everyday clinical practice according to G&P. This is partly because the hand is not yet sufficiently ossified in young children and already too ossified in near adults. Hence, gender-based subgroups were formed for the mid-90% of the age percentiles. The 5th and 95th age percentiles required for this purpose were calculated through the analysis of the chronological age (CA) of every BA exam in the same center between 2014 and 2024 (6183 examinations, 3178 exams of male patients).

### Determination of bone age

All X-ray images used for the study were taken on an Axiom Aristos FX (Siemens Healthcare) without a scatter grid, with a 0.1 mm copper filter. The left hand including the wrist was always examined in posterior-anterior projection.

All four vendors of AI-assisted BA determination according to G&P certified for the European market were asked to participate in this study: three vendors agreed to participate in the study (Visiana, ImageBiopsy Lab, Gleamer), no response was received from the fourth vendor (VUNO).

All 306 radiographs were examined by three readers (two pediatric radiologists, D.G. and A.B.B., with 7 and 4 years of experience, respectively, and a pediatric endocrinologist, R.P., with 30 years of experience in pediatric BA determination) in independent sessions to determine the manual BA. BA was determined using the atlas of G&P [2]. If the ages of the carpals and epiphyses were different, the age of the epiphyses was preferred [15, 16]. The mean value of the BA estimates by three readers was used as a reference for further evaluation (“ground truth”).

Likewise, all 306 images were submitted for evaluation to the included AI programs for BA determination: BoneXpert (v. 3.2.2), PANDA (v 1.13.21), and BoneView (v. 2.3.1.1). BoneXpert and PANDA were available as local standalone apps, whereas BoneView evaluation was cloud-based. BoneView rejects analysis for a CA below 3 years and returns every BA above 17 years of age as “≥ 17 y”.

### Statistics

The evaluation was conducted using RStudio Version 2023.06.2 (PBC). Mean values with standard deviations (SD) or median with interquartile range (IQR) were given as appropriate. The mean error, the mean absolute error (MAE), and the root mean squared error (RMSE) were determined as performance parameters. A Bland–Altman plot was used to determine the fixed bias and the limits of agreement as a measure of the variation between AI and human readers. The mean SD between the three AI programs and between the three readers was determined as root mean square ${RMS}=\,\sqrt{\frac{1}{n}\left({{{SD}}_{1}}^{2}+\,{{{SD}}_{2}}^{2}+\,\ldots +{{{SD}}_{n}}^{2}\right)}$ where ${{SD}}_{i}$ is the standard deviation of the three bone age values of the i-th patient obtained using the three AI programs or from the three readers. A comparison of the SDs of the AI programs with the SDs of the human readers was performed by a two-tailed Wilcoxon signed-rank test. Next, the assumption of a constant difference between the methods, i.e., zero slope in the Bland–Altman plots, was relaxed. The differences were regressed on the averages, and the results were used to obtain prediction intervals of the differences between BA as estimated by AI and the ground truth. Additionally, prediction equations and prediction intervals of the ground truth, given an AI measurement, were derived [17]. A two-tailed Student’s t-test was used to test if the slope in the prediction equation was equal to one (equivalent to constant mean error or no slope in the Bland-Altman plot). The R package “MethComp” was used for these computations. The Friedman rank sum test was applied to squared differences between BA as estimated by AI and the ground truth to compare the accuracy of the different AI methods. Interreader variabilities for the expert readers for this cohort were reported in literature [15]. Differences in the central tendency were determined using the student t-test, with the significance level set at 0.05.

## Results

### Patient cohort

Hand radiographs of 306 patients (153 females) were included. The median age was 9.4 years (IQR 5.2–13.5) for males and 9.6 years (IQR 5.2–13.5) for females. The subgroup of the most common age range (5–95th percentile of CA) had ages spanning 4.8–15.5 years for females and 4.9–17.0 years for males and resulted in 206 patients (109 males). Demographic data of the patient cohort were also published prior [15] (Supplementary Table 1).

### Determination of bone age

The median G&P BA estimated by the expert readers was 9.3 years (IQR 4.3–13.2) for males and 9.6 years (IQR 4.5–13.0) for females. None of the radiographs were considered unreadable by the expert readers or PANDA. Meanwhile, BoneXpert rejected the evaluations of seven of 306 (2.3%) examinations due to the software’s internal quality control, and BoneView rejected 62/306 (20.3%) examinations that were outside the CA approved by the software (35 children with a CA < 3 years and 27 patients with a BA ≥ 17 years).

### Performance in the whole cohort

**Fig. 1:**
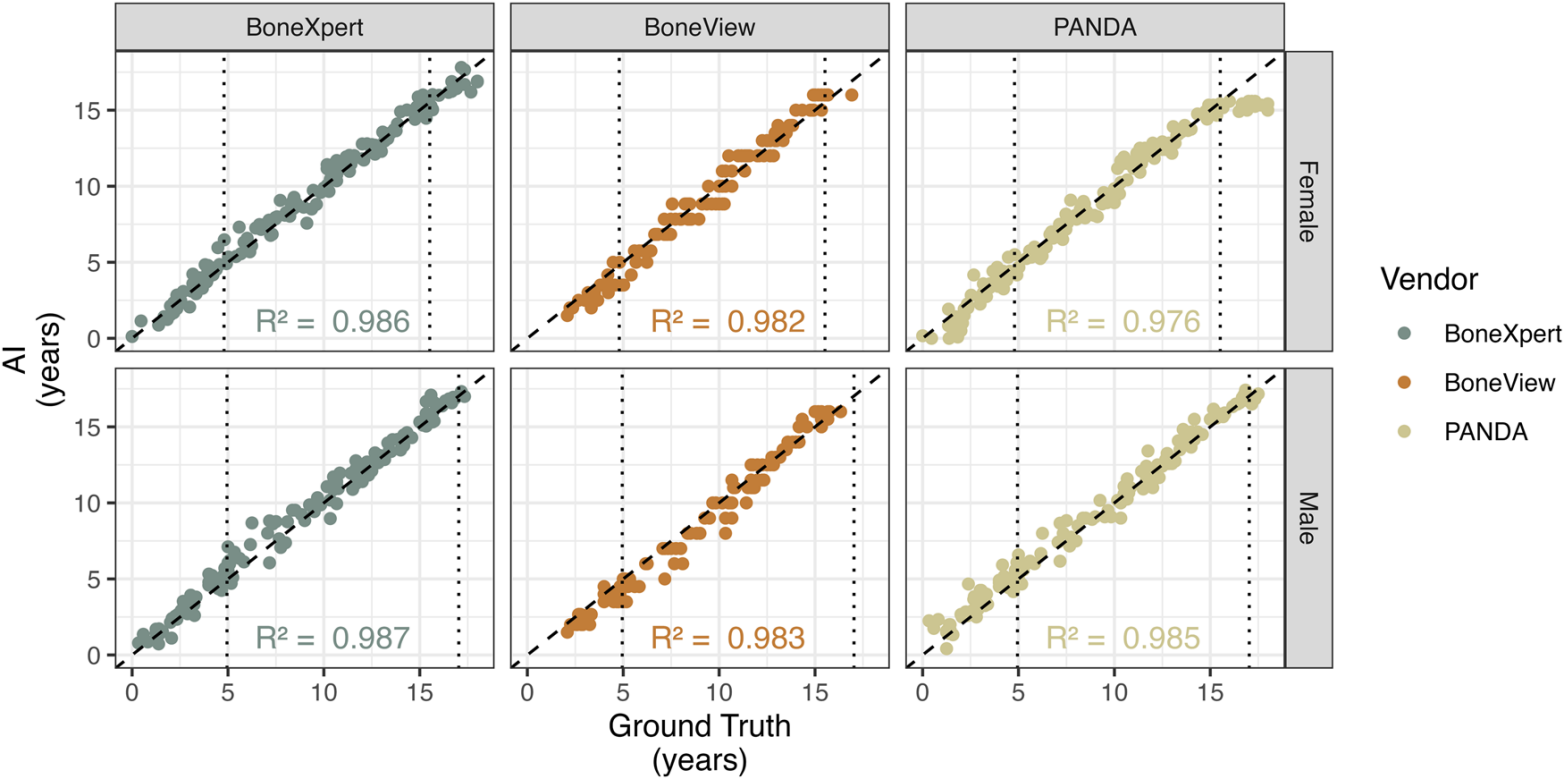
Correlation for bone age estimation between ground truth (determined via expert readers) and three AI-based programs. The area between the dashed vertical lines corresponds to the age collective that accounts for 90% of the bone age determinations in the study center. Please note: BoneView estimates exclude children with a chronological age below 3 years and a bone age above 17 years. Thus, the correlation coefficient does not reflect BoneView’s ability to analyze the entire cohort

The correlation between AI and the expert readers regarding BA estimation was very high across all three programs at *R*^2^, between 0.976 and 0.987 (Fig. 1). The standard deviation between the three AI programs was lower on average than that between the three expert readers (0.52 years vs. 0.62 years, *p* < 0.01). Across the entire age range, BoneXpert had a lower RMSE (0.62 years) compared to BoneView (0.69 years) and PANDA (0.75 years) (Table 1). Notably, BoneView rejects analysis with CA below 3 years, thus the parameters for BoneView do not reflect completely the same cohort as opposed to BoneXpert and PANDA.

**Table 1:** Prediction error for bone age in the entire patient population (see * for BoneView) for the three software programs

	BoneXpert (*n* = 299)			BoneView (*n* = 244)*			PANDA (*n* = 306)		
	Female	Male	Total	Female	Male	Total	Female	Male	Total
RMSE (years)	0.58	0.66	0.62	0.60	0.69	0.65	0.79	0.72	0.75
MAE (years)	0.46	0.51	0.48	0.49	0.52	0.51	0.58	0.54	0.56
Mean error (years)	0.09	0.30	0.19	−0.07	−0.23	−0.15	−0.21	0.28	0.04

RMSE, MAE and mean error were computed based on the differences between the bone ages estimated by each AI program and the means of the bone ages obtained from the three human readers (ground truth)

*RMSE* root mean squared error, *MAE* mean absolute error

* BoneView, in contrast to BoneXpert and PANDA, rejects analysis if the chronological age is below 3 years and bone age is above 17 years. Due to the resulting 20.3% dropouts, the table does not reflect BoneView’s ability to analyze the entire patient population

### Performance in the subgroup

**Fig. 2:**
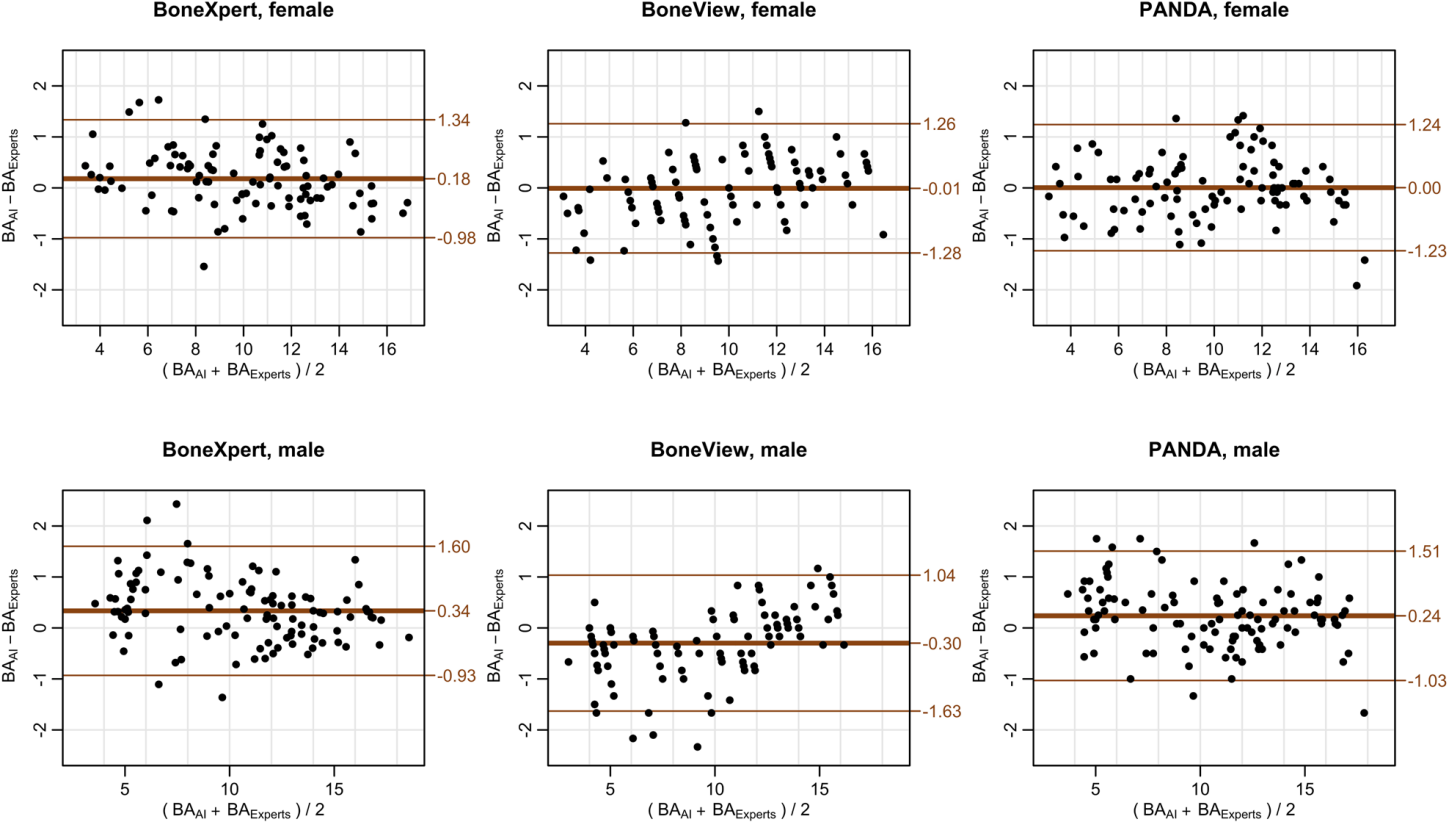
Bland–Altman plots the estimation accuracy of the three programs for artificial intelligence (AI) supported bone age determination for the subgroup of the common age range (age given in years)

In the subgroup comprising 90% of the usual study population at the study center, PANDA had a slightly lower RMSE (0.65 years) than BoneXpert (0.66 years) and BoneView (0.68 years) (Table 2 and Fig. 2). The dropout rate was 1.5%, 4.4% and 0% for BoneXpert, BoneView and PANDA respectively. The mean error of 0.13 years was also lower for PANDA than that for BoneXpert (0.26 years) and BoneView (−0.15 years). The differences between the squared residuals of the three BA estimates were not significant (*p* = 0.15).

**Table 2:** Extent of prediction error for bone age in the subgroup of the common age range for bone age determination

	BoneXpert (*n* = 203)			BoneView (*n* = 197)			PANDA (*n* = 206)		
	Female	Male	Total	Female	Male	Total	Female	Male	Total
RMSE (years)	0.60	0.71	0.66	0.63	0.72	0.68	0.62	0.68	0.65
MAE (years)	0.47	0.56	0.52	0.51	0.53	0.52	0.48	0.52	0.50
Mean error (years)	0.18	0.34	0.26	−0.01	−0.28	−0.15	0.00	0.24	0.13

*RMSE* root mean squared error, *MAE* mean absolute error

### Conversion from AI to expert reader

**Fig. 3:**
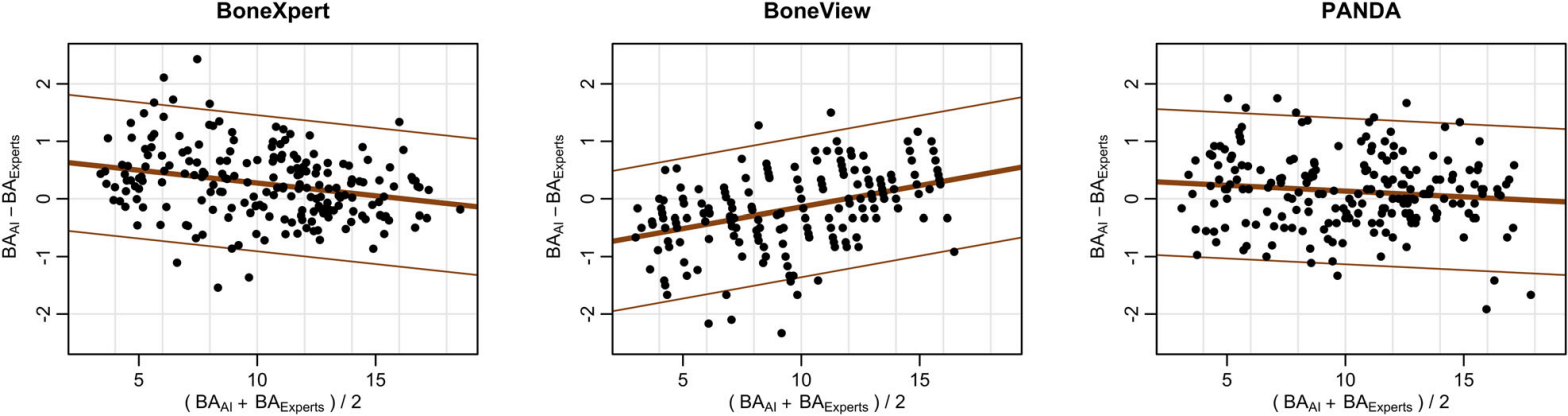
Bland–Altman plot for the measurement of bone age by software and expert reader, assuming a linear dependence of the measurement deviation on increasing mean value (age given in years)

For the subgroup within the most common age span, conversion factors were calculated to adjust the BA estimated by the AI to match that of the expert readers. The mean error for BoneXpert and BoneView did not remain constant across the entire BA range (*p* < 0.001 for both methods). Assuming a linear relationship, there was a slight decrease in the mean error for BoneXpert and an increase for BoneView (Fig. 3).

These changes in the mean error were not significant for PANDA (*p* = 0.09). Using the data shown in Table 3, the ground truth can be estimated using BA_experts_ = intercept + slope * BA_ai_, with the prediction interval ± 2 * (prediction standard deviation) around this estimated value. The prediction standard deviation (and thus the prediction limits), which measures the uncertainty of the prediction of the ground truth, was comparable for BoneView and BoneXpert (0.60 years) and slightly larger for PANDA (0.64 years).

**Table 3:** Parameters for converting the bone age (in years) estimated by AI into the bone age estimated by expert readers (part: “Conversion from AI to Ground truth”)

	Conversion from AI to ground truth				Bland–Altman plot		
	Intercept	Slope	SD	*p*-value*	Intercept	Slope	SD
BoneXpert	−0.735	1.045	0.604	< 0.001	0.718	−0.044	0.591
BoneView	0.784	0.937	0.597	< 0.001	−0.810	0.065	0.616
PANDA	−0.338	1.020	0.640	0.094	0.335	−0.020	0.634

These parameters are the intercept and slope of the conversion line and the residual (or prediction) standard deviation. They were derived from the corresponding parameters obtained by regressing the differences between the methods on the averages (part: “Bland-Altman plot”) using the previously published formulas: −a/(1 + b/2), (1 − b/2)/(1 + b/2), and s/(1 + b/2), where a, b and s denote the intercept, slope and the residual standard deviation in the Bland-Altman plot, respectively [17]. The Bland-Altman plots are shown in Fig. 3

* *p*-value to reject the null hypothesis where slope = 1

## Discussion

This study is the first to compare three of the four programs for BA determination based on the atlas of G&P that have been approved for the European market as medical devices.

All three programs analyzed in the present study - BoneXpert, PANDA, and BoneView -  predict BA according to G&P reliably and with similar quality with an MAE of 0.48–0.56 years. However, it should be mentioned that BoneView does not analyze the age group up to 3 years of CA, which is particularly difficult to evaluate but is also rarely used in clinical practice. Also, BoneView returns every BA above 17 years as “≥ 17 y”, due to forensic considerations, resulting in a rejection rate of one-fifth of the whole cohort.

In the subgroup containing the main age range, the MAE even fell between 0.50 and 0.52 years. Notably, the mean standard deviation among the three AI programs was lower than that of the human readers, consistent with findings in the literature and indicating a quality feature [18]. In previous validation studies, all three programs showed excellent agreement between AI-based and expert reader assessments. The analyzed programs also showed similar deviations of BA as estimated by the AI as compared to human readers in previous studies: BoneXpert had a MAE of 0.34 years and an RMSE of 0.45 years [5], BoneView had a MAE of 0.49 years [19]. To the authors’ best knowledge, only one recent study evaluated more than one AI-supported program for BA determination concurrently: BoneXpert and Med-BoneAge [14]. No significant differences between the two AI programs and expert readers were found [14]. Med-Bone Age was not included in the current study due to a lack of response from the vendor.

The differences between the AI programs are more apparent in the details: While BoneXpert produced the best results over the entire age range of 1–18 years, PANDA showed an even smaller deviation from the expert readers evaluation for the main age range, which we defined as 90% of all BA examinations in clinical practice. However, outside this age range, the results of PANDA were worse. Therefore, it is not surprising that the main age range determined in this study largely coincides with the age range of intended use of PANDA (> 3 years and < 16 years for females and < 17 years for males). While BoneView rejects examinations beyond the recommended CA before the analysis, PANDA analyzes every patient (and, in principle, even X-rays not meant for G&P BA analysis, such as chest X-rays) and leaves it to the user to validate the correct input data. This is a trade-off for the high acceptance rate of PANDA and BoneView. Nevertheless, for cases with a CA outside the intended use range, a warning is issued by PANDA in the findings report. Considering the ceiling effect for BA beyond the recommended CA (Fig. 1), the results for PANDA in near adults must be interpreted with caution.

Additionally, PANDA analyses have been shown to be quite robust against non-straight posterior-anterior projections of the hand [20]. In contrast to BoneView and PANDA, BoneXpert has an internal quality control for hand radiographs. In the study dataset, 2.2% of the examinations were rejected.

The clinical relevance of a mean absolute deviation of approximately half a year is unclear. This is further emphasized by the fact that human readers in this study and the literature show a similar standard deviation [18]. Furthermore, the extent to which mean deviations of weeks to a few months, as observed among the three programs studied, are significant for clinical practice in the context of such high natural variability remains speculative. Without the adjustments described in Table 3, the proportional bias in BoneXpert would result in a tendency to overestimate and, in BoneView, a tendency to underestimate in prepubescent children.

The software programs also differ in their requirements for the selection of X-ray images and the patient population: Of the three AI software programs analyzed, BoneXpert is the most used and established software and has been validated in many studies as well as different ethnicities and diseases [13]. Moreover, BoneXpert was validated in an actual Caucasian population [21]. In contrast to the other software, BoneXpert was validated for BA determination based on both G&P and the Tanner–Whitehouse method. It can analyze both left- and right-hand radiographs in the anterioroposterior view [13]. BoneXpert is unique in having an intended use not only as an AI-assist tool but also as an AI-replace tool. The additional option of determining a “bone health index” also extends the sheer BA determination by the AI software (“AI-expand”) [13]. BoneView is the newest AI software and can analyze anteroposterior views and radiographs of the left or right hand [19].

The authors acknowledge several limitations of the study: The study population comprised patients with suspected or proven growth failure from a single center. Since some pathologies affect not only BA but also bone morphology (e.g., achondroplasia), a center with a different patient composition may achieve different results. The cohort studied was comprised of Caucasians only, so the results may not be generalized to other ethnicities.

The economic requirements of the programs were not compared as part of the study, as pricing depends on both the volume of analysis and the negotiating conditions of the clinical facilities, such as whether other programs from the same provider are already in operation on-site. BoneView rounds each BA to an age category given in the G&P atlas, while PANDA and BoneXpert give continuous values. Since even the gold standard, the mean value of three expert readers, does not correspond exactly to G&P stages, the performance of BoneView without rounding to G&P age categories could, in theory, be higher (Supplementary Tables 2 and 3). However, in the commercial version, only the rounded values are available. Most importantly, the “gold standard,” the expert reader, can also be flawed. Inter- and intra-reader variability in the G&P atlas, averaging the BA estimates of three expert readers, was used to mitigate this limitation.

In summary, all three AI-supported programs showed a high degree of accuracy in determining the G&P BA. Differences emerge in the details, particularly in infants and near adults.

## Supplementary information

ELECTRONIC SUPPLEMENTARY MATERIAL

## Abbreviations

AI: Artificial intelligence
BA: Bone age
CA: Chronological age
CE: Conformité Européenne
G&P: Greulich and Pyle
IQR: Interquartile range
MAE: Mean absolute error
RMSE: Root mean squared error
SD: Standard deviation
