# Supplementary material for: A critical comparative study of the performance of three AI-assisted programs for bone age determination
Source: Eur Radiol. 2024 Nov 5;35(3):1190–6. doi: 10.1007/s00330-024-11169-6 (PMC11835896; doi:10.1007/s00330-024-11169-6)
Supplement: Supplementary file 1 — ELECTRONIC SUPPLEMENTARY MATERIAL [file 330_2024_11169_MOESM1_ESM.pdf]

A critical comparative study of the performance of three AI-assisted programs for bone age determination

ELECTRONIC SUPPLEMENTARY MATERIAL

Eur Radiol (2024) Pape J, Rosolowski M, Pfäffle R, Beeskow AB, Gräfe D.

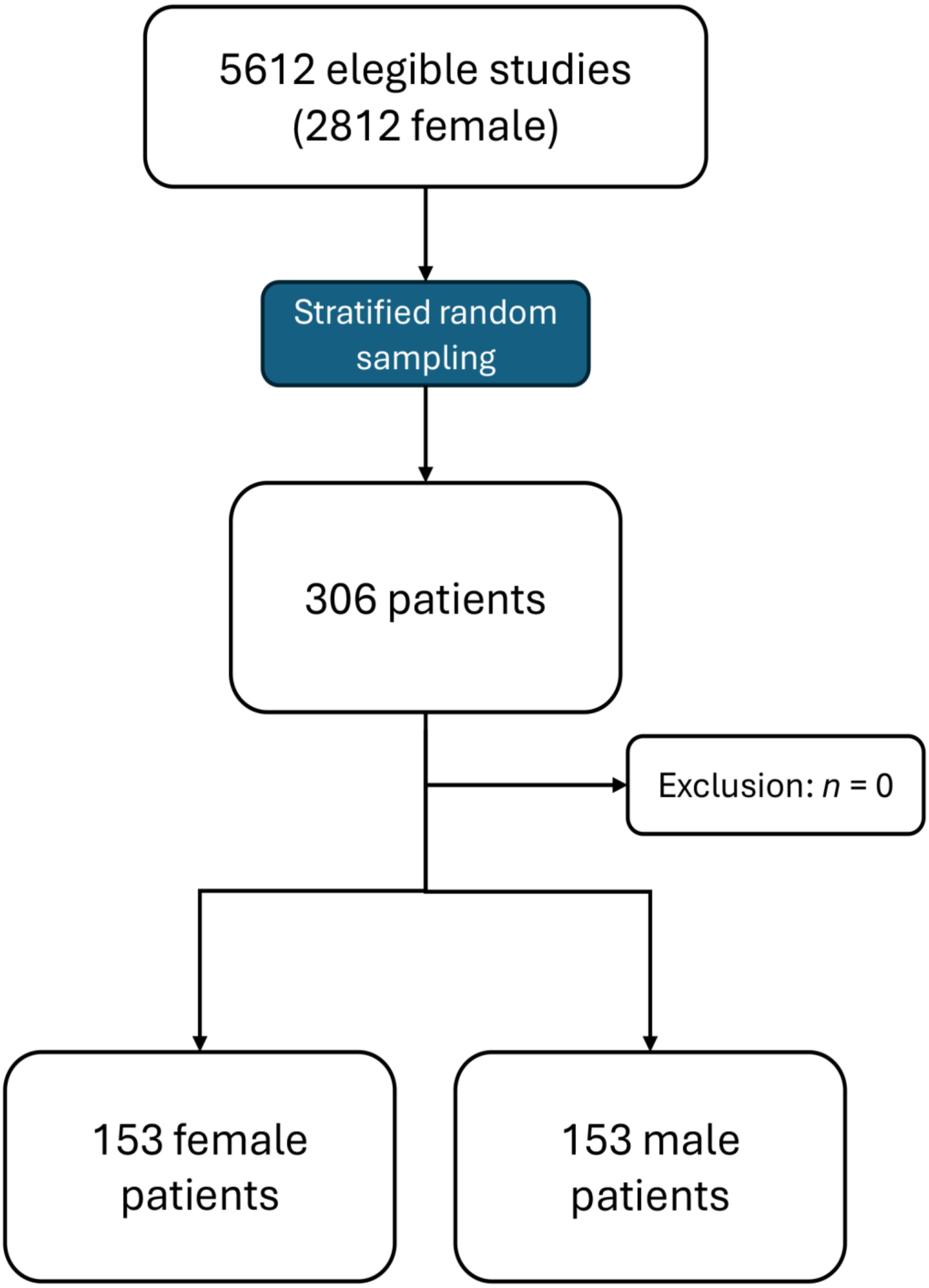

Supplemental Fig 1 Inclusion flowchart for the study cohort, published in (15).

Supplemental table 1: Demographic data of the study population, published in (15).

	**Study population**
**Patients (#)**	306
**Gender**	153 Female
**Age (y)**	9.5 (± 4.9)
**Height (cm)**	131.1 (± 30.0)
**Weight (kg)**	35.3 (± 23.2)
**BMI (kg/m^2)**	18.3 (± 6.3)

BMI: Body mass index

Supplemental table 2 Prediction error for bone age in the entire patient population (see * for BoneView) for the three software programs, without BoneView rounding to Greulich and Pyle age categories (not available in the commercial version)

	**BoneXpert** (n=299)			**BoneView *** (n=244)			**PANDA** (n=306)		
	Female	Male	*Total*	Female	Male	*Total*	Female	Male	*Total*
**RMSE** (y)	0.58	0.66	*0.62*	0.60	0.69	*0.65*	0.79	0.72	*0.75*
**MAE** (y)	0.46	0.51	*0.48*	0.49	0.52	*0.51*	0.58	0.54	*0.56*
**Mean Error** (y)	0.09	0.30	*0.19*	-0.07	-0.23	*-0.15*	-0.21	0.28	*0.04*

RMSE: Root mean squared error; MAE: Mean absolute error.

* BoneView, in contrast to BoneXpert and PANDA, rejects analysis if CA is less than 3 years. Thus, the table does not reflect BoneView's ability to analyse the entire patient population (i.e. no children below 3 years chronological age)

Supplemental table 3 Extent of prediction error for bone age in the subgroup of the common age range for bone age determination without BoneView rounding to Greulich and Pyle age categories (not available in the commercial version)

	**BoneXpert** (n=203)			**BoneView** (n=197)			**PANDA** (n=206)		
	Female	Male	*Total*	Female	Male	*Total*	Female	Male	*Total*
**RMSE** (y)	0.60	0.71	*0.66*	0.51	0.69	*0.63*	0.62	0.68	*0.65*
**MAE** (y)	0.47	0.56	*0.52*	0.45	0.51	*0.48*	0.48	0.52	*0.50*
**Mean Error** (y)	0.18	0.34	*0.26*	-0.03	-0.25	*-0.15*	0.00	0.24	*0.13*

RMSE: Root mean squared error; MAE: Mean absolute error.
